# Potential molecular consequences of transgene integration: The R6/2 mouse example

**DOI:** 10.1038/srep41120

**Published:** 2017-01-25

**Authors:** Jessie C. Jacobsen, Serkan Erdin, Colby Chiang, Carrie Hanscom, Renee R. Handley, Douglas D. Barker, Alex Stortchevoi, Ian Blumenthal, Suzanne J. Reid, Russell G. Snell, Marcy E. MacDonald, A. Jennifer Morton, Carl Ernst, James F. Gusella, Michael E. Talkowski

**Affiliations:** 1 Centre for Brain Research, School of Biological Sciences, The University of Auckland 1010, New Zealand; 2 Molecular Neurogenetics Unit, Center for Human Genetic Research, Massachusetts General Hospital, Boston, Massachusetts 02114, USA; 3 Program in Medical and Population Genetics, Broad Institute of M.I.T and Harvard, Cambridge, Massachusetts 02143, USA; 4 McDonnell Genome Institute, Washington University School of Medicine, St. Louis, Missouri 63108, USA; 5 Department of Neurology, Harvard Medical School, Boston, Massachusetts 02115 USA; 6 Department of Physiology, Development and Neuroscience, University of Cambridge, Downing Street, Cambridge CB2 3DY, United Kingdom; 7 Department of Psychiatry, McGill University, Montreal, Quebec ON H4H 1R3, Canada; 8 Department of Genetics, Harvard Medical School, Boston, Massachusetts 02115 USA; 9 Psychiatric and Neurodevelopmental Genetics Unit, Center for Human Genetic Research, Massachusetts General Hospital, Boston, Massachusetts, 02114 USA

## Abstract

Integration of exogenous DNA into a host genome represents an important route to generate animal and cellular models for exploration into human disease and therapeutic development. In most models, little is known concerning structural integrity of the transgene, precise site of integration, or its impact on the host genome. We previously used whole-genome and targeted sequencing approaches to reconstruct transgene structure and integration sites in models of Huntington’s disease, revealing complex structural rearrangements that can result from transgenesis. Here, we demonstrate in the R6/2 mouse, a widely used Huntington’s disease model, that integration of a rearranged transgene with coincident deletion of 5,444 bp of host genome within the gene *Gm12695* has striking molecular consequences. *Gm12695*, the function of which is unknown, is normally expressed at negligible levels in mouse brain, but transgene integration has resulted in cortical expression of a partial fragment (exons 8–11) 3’ to the transgene integration site in R6/2. This transcript shows significant expression among the extensive network of differentially expressed genes associated with this model, including synaptic transmission, cell signalling and transcription. These data illustrate the value of sequence-level resolution of transgene insertions and transcription analysis to inform phenotypic characterization of transgenic models utilized in therapeutic research.

Fundamental questions in human biology and therapeutic development have been evaluated using transgenic model organisms, typically generated by pronuclear injection. Despite its widespread use, relatively little is known concerning the detailed impact of random transgene integration on the host genome as a result of pronuclear injection, although there has been documented evidence of position effect variegation and insertional mutations disrupting endogenous genes^1^. Conventional methods to characterize transgene integrations (e.g. fluorescence *in situ* hybridization [FISH] or Southern blotting) lack the resolution to determine the integration site, and sequence-level integrity of both the transgene and host genome.

We have shown previously that customizations in next-generation sequencing methods can deliver unambiguous localization of transgenic integration sites and characterization of the transgene architecture at nucleotide level resolution. We applied these methods to a number of Huntington’s disease (HD) model organisms^2^, including the widely studied R6/2 transgenic mouse model^3^. This line was one of a series created in 1996 by pronuclear injection of a 1.9 kb fragment from the 5′ end of human *HTT,* containing the first exon with a ~130 unit CAG trinucleotide repeat expansion. In the R6/2 mouse, an amino-terminal polyglutamine-containing fragment of huntingtin is expressed and leads to rapid and severe neurological abnormalities at a younger age than other N-terminal *HTT*-fragment transgenic mouse models, making this mouse line attractive for therapeutic studies.

Using a series of targeted and whole-genome sequencing approaches, we previously revealed significant structural rearrangement of the transgene in R6/2 genomic DNA at nucleotide resolution, including excision/insertion and inversion events that left a single copy of the 5′ region and exon 1 CAG expansion region downstream of a 168 bp insertion of bacterial DNA and of intron 1 sequence, along with additional rearrangement of the upstream and intron 1 sequences (Fig. 1, see Chiang *et al*.^2^ for complete details, GenBank: KF990992.1). This structure suggests the R6/2 transgene integration event involved fragments derived from at least three copies of the original injected *HTT* fragment, as originally speculated by the authors who created the model^3^ and suggested by previous analyses^4^. However, we also found complex inversions, as well as head-to-tail and tail-to-head concatenations. This entire segment was inserted, with coincident deletion of 5,444 bp of host DNA, within intron 7 of *Gm12695* in mouse chromosome 4. The transgene inserted in antisense orientation to *Gm12695* transcription, deleting a segment predicted to harbour multiple transcription factor binding and DNaseI hypersensitivity sites that encompass two regions of greater than 90% identity across mammals (Fig. 1). Here, we further these studies by assessing the transcriptional impact of the transgenic integration in the R6/2 mouse.

## Results

**Figure 2:**
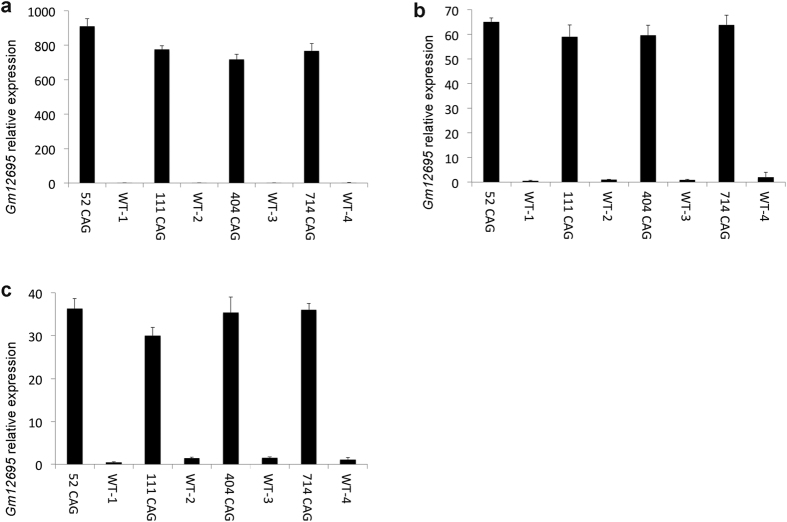
Dysregulation of *Gm12695* expression in R6/2 brain. Expression of *Gm12695* mRNA sequences in (**a**) cortex, (**b**) hippocampus and (**c**) striatum of four independent R6/2 transgenic mice expressing either 52, 111, 404 or 714 CAG repeats (which display differing phenotypic severity), and four age and sex-matched littermates that do not carry the transgene, was measured using quantitative PCR. All brain regions in transgenic R6/2 mice showed dramatically increased expression of *Gm12695* sequences above low or negligible expression levels in wild-type. Data are represented as mean ± STDEV.

To determine whether the transgene integration event in the R6/2 genome had an effect on expression of *Gm12695* in cortical tissue, we initially used a quantitative RT-PCR approach. We found that *Gm12695* is expressed at negligible levels in the cortex of non-transgenic (wild-type) littermates but showed evidence of dramatically increased levels, greater than 30-fold, in the corresponding brain tissues of R6/2 mice. There were at least two potential explanations for this increase; 1) the integration of the transgene within the noncoding intronic sequence of *Gm12695* profoundly disrupts its normal regulation, resulting in altered expression of this gene in these regions of the brain, or 2) the increased expression of *Gm12695* is an indirect consequence of the expression of the R6/2 transgene, as a reaction to the pathogenesis/toxicity caused by expression of the polyglutamine fragment. To distinguish between these options, we examined further mouse lines that are descended from the original R6/2 mouse line but show marked differences in their severity of phenotype due to substantial changes in the length of the CAG trinucleotide repeat. The repeat is known to be unstable in these mice^3^ both in somatic cells and in the germline, hence selective breeding has enabled an allelic series to be generated harbouring different CAG repeat lengths^5^, as determined by Laragen Inc.^6^. Mice with considerably longer repeats (404 CAGs, 714 CAGs) or shorter repeats (52 CAGs) display a less severe phenotype than the parental R6/2 strain (111 CAGs) from which they were generated, with a delay in symptom progression and prolonged survival^5^,^7^. We reasoned that if *Gm12695* expression was a consequence of pathogenesis/toxicity induced by the expression of the polyglutamine fragment, its expression would vary across each model harbouring different CAG repeat lengths. If, on the other hand, its expression were independent of polyglutamine fragment pathogenesis/toxicity, we would expect its expression to remain unchanged across each of these models. We used the Pfaffl analysis method^8^ with primers spanning exons 8 and 9 of *Gm12695* and normalized to the reference gene *Atp5b.* We found that *Gm12695* is highly expressed in all four CAG repeat expansion mice (52, 111, 404 and 714 CAG repeats), in three different brain regions (cortex, hippocampus, striatum) (Fig. 2a,b,c), suggesting that the increased expression of *Gm12695* is not a consequence of pathology/toxicity caused by expression of the transgene-encoded polyglutamine fragment. Expression analysis in two peripheral tissues, heart and liver, demonstrated the same aberrant expression of *Gm12695* in R6/2 (Supplementary Fig. S1).

**Figure 3:**
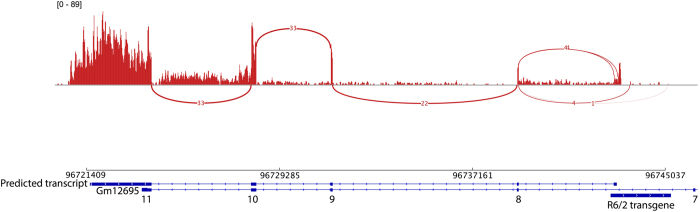
Identification of a partial *Gm12695* transcript spliced downstream of a 3′ segment of R6/2 transgene. Integrated Genome Viewer display of combined RNA sequencing data from all three R6/2 libraries. The Sashimi plot depicts splice junctions as arcs between the R6/2 transgene and exon 8, and between other exons of the *Gm12695* transcript on the sense strand (reverse strand). Numbers on the arcs illustrate the reads covering splice junctions. Maximum read depth coverage is 89. The bottom track illustrates a novel transcript predicted by Trinity (predicted transcript) as a result of transgene integration, the known *Gm12695* transcript and the R6/2 transgene at the integration site. The chromosomal coordinates are derived from Supplementary Table S1.

To explore the consequent alterations to normal transcription, both locally and globally of the transgene integration event, we performed RNA sequencing (RNAseq) on cortical brain tissue from three R6/2 mice (160 CAG+/−5) obtained from the Jackson Laboratory and three littermate controls. RNAseq revealed that the normal transcript structure of *Gm12695* was significantly altered in R6/2 samples. Specifically, a portion of the 3′ end of the transgene (on the reverse strand) spliced to exons 8–11 of *Gm12695* (sense strand) were joined, creating a possible open reading frame (see Fig. 3, Supplementary Fig. S2). This is consistent with the Q-PCR data, which targeted transcription from exons 8 and 9 of *Gm12695.* The transcriptome assembly tool, Trinity^9^, also predicted this transcript. The majority of transcript sequence reads were aligned to a region on the reverse strand of the transgene spanning chr4:96,743,050–96,743,250, which overlaps with bacterial DNA that was integrated into the transgene structure following pronuclear injection (Supplementary Table S1). Relatively few transcript reads aligned over the rest of the transgene on the reverse strand, including the CAG repeat region due to secondary structure of the RNA and the intractability of aligning long repetitive sequences (Supplementary Fig. S4).

Minimal reads mapped to the forward strand of the transgene (antisense strand of *Gm12695*) (Supplementary Figs S3 and S5). However, downstream of the transgene insertion, in intron 7 of *Gm12695* there was remarkably high levels of expression (Supplementary Fig. S3). This region is predicted to contain repeat elements including a long terminal repeat (LTR), MLT1A on the reverse strand and a long interspersed nuclear element (LINE), L1Md_T on the forward strand (Supplementary Fig. S5). Neither splice junctions nor mapped paired-end reads spanning both the transgene and repeat elements in *Gm12695* were detected (Supplementary Figs S2 and S3), hence no new open reading frames were created. However, it is possible that transgene integration has activated expression of these elements, which are not normally transcribed in control mice. As predicted, we did not detect reads matching *Gm12695* or the R6/2 transgene in the control mice on either strand.

To analyse whether expression of the transgene and partial expression of *Gm12695* were associated with alteration of other gene networks in the R6/2 model, we used differential expression analysis to compare R6/2 and control samples. We detected 19,514 expressed genes among which 65 were significantly differentially expressed at adjusted p-value < 0.05 and 841 were differentially expressed at nominal p-value < 0.05 (Supplementary Tables S2 and S3). Pathway and gene ontology (GO) term overrepresentation analysis revealed that R6/2 down-regulated genes at nominal p < 0.05 were associated with terms related to neurobiology including “neurological system process”, “synaptic transmission”, “neuropeptide signalling pathway”, “synapse”, “voltage-gated ion channel activity”, “cell-cell signalling” and “calcium signalling pathway”, whereas R6/2 up-regulated genes at nominal p < 0.05 were enriched for terms associated with “transcription” (Supplementary Table S4). Not surprisingly, the partially transcribed *Gm12695* (sense strand) was the most significant differentially expressed transcript (adjusted p-value = 6.66e-28), whereas differential expression of the transgene on the corresponding forward strand ranked 40^th^ (adjusted p-value = 1.86e-2; Supplementary Table S3).

We also investigated *Gm12695* expression using RNA sequencing data from heterozygous *Htt* CAG repeat expansion knock-in mice^10^, genetic replicas of the HD mutation. Members of this allelic knock-in series harbouring increasing size CAG repeat tracts (often named Q20, Q80, Q92, Q111 generated by Wheeler *et al*.^11^ and Q140, Q175 generated by Menalled *et al*.^12^,^13^) exhibit a variety of disease-allele associated phentoypes^12^,^14^,^15^,^16^,^17^. We found no evidence of *Gm12695* expression across any of the expanded CAG repeat lengths, tissue types (both neuronal and non-neuronal) and ages for this allelic series (Supplementary Fig. S6). We also identified a recent HD post-mortem RNA sequencing dataset^18^ which explored global transcriptional dysregulation in HD and control prefrontal cortex. We found the human orthologue of this gene (*C1orf87*) to be upregulated in HD cortex, however this effect was removed when the dataset was adjusted for clinical covariates such as CAG repeat length and residual age of onset. These data both support the notion that aberrant expression of *Gm12695* is specifically related to the transgene insertion event in R6/2 and is not an indirect consequence of HD pathology.

## Discussion

Using Q-PCR and RNAseq we have shown the transcriptional impact of transgenic integration in a fragment model of Huntington’s disease, the R6/2 mouse model, one of the most widely studied models of the disease. Initial elucidation of the genomic insertion site, into the conserved gene *Gm12695,* revealed complex structural rearrangement, including a 5.4 kb deletion of host *Gm12695* sequence. Q-PCR studies targeted downstream of the insertion site revealed this gene to be abnormally highly expressed in R6/2 brain across mice with varying phenotypic severity. This suggested that the increased expression of the host gene sequences was not an indirect reaction to the pathogenesis/toxicity caused by expression of the polyglutamine fragment, but rather due to the transgene integration event.

To clarify the impact of the transgene and concomitant deletion of the host sequence, we used RNAseq to reveal transcriptional activation of the downstream mouse *Gm12695* exons 8–11, as well as expression of an upstream LINE sequence on the opposite strand. These changes in gene expression are associated with differentially expressed gene networks in R6/2 mice, including synaptic transmission, cell signalling and transcription. Thus, the integration of the transgene into intron 7 of *Gm12695* appears to substantially disrupt its normal regulation, resulting in dysregulated expression of a conserved gene that is not normally expressed (or expressed at very low levels) in mouse brain. Transcriptional analysis of an HD mouse knock-in allelic series^11^,^12^,^13^ and a human post-mortem dataset^18^ did not reveal significant dysregulation of *Gm12695* or its human orthologue *C1orf87*, supporting the finding that abnormal regulation of *Gm12695* is a consequence of transgene integration in R6/2. Dysregulation of this gene has not been a focus of previous gene expression studies in this R6/2 mouse model. This may be because the gene is largely uncharacterized with unknown function, although the gene has been implicated in amyloid beta pathology in Alzheimer’s disease^19^,^20^.

*Gm12695,* like its conserved human orthologue, *C1orf87*, encodes an anonymous EF-hand protein of which little is known. It is not clear whether this abnormal expression of *Gm12695* is due specifically to the insertion of the transgene sequence, the deletion of the 5.4 kb host *Gm12695* sequence, or the combination of the two. This may be elucidated using a CRISPR-based approach to model the integration event in neuronal or induced pluripotent stem cells. We also cannot definitively disentangle whether this expression change contributes to any phenotypes shared by R6/2 and its sub-lines, or whether it acts as a modifier on the sensitized background of R6/2 to make the model more severe than other N-terminal fragment models.

These results suggest that it is advisable to determine the genomic integration site and structural architecture of introduced transgenes to understand fully the consequences of transgenesis. This can be readily accomplished by next-generation sequencing methods as we have reported with transgenic mouse and sheep models utilized in HD research^2^. Furthermore, the same methods can be applied to achieve the necessary high-resolution structural characterization for other genome integration phenomena, including viral insertions or the introduction of reprogramming transcription factors in induced pluripotent stem cell models. Capitalizing on the capability of current genomics technologies to characterize disease models in advance of costly functional studies may provide a valuable guide to choosing individual lines best suited for biological interpretation and therapeutic development.

## Methods

### Samples

R6/2 mouse mRNA was prepared from fresh frozen brain tissue (cortex, hippocampus and striatum) from either in-house colonies (R6/2 mice and their wild-type littermates) at the University of Cambridge or from tissue purchased from the Jackson Laboratory. The Jackson Laboratory is fully accredited by the Association for Assessment and Accreditation of Laboratory Animal Care International (AAALAC International). This study was carried out in accordance with the recommendations of the United Kingdom 1986 Animals (Scientific Procedures Act). The protocol was approved by the Ethical Review committee of the University of Cambridge. RNA was prepared for Quantitative PCR and RNA sequencing from ~30 mg of brain tissue, homogenized using QIAzol lysis reagent (Qiagen) in a Qiagen TissueLyser (2 × 2 min at 20 Hz). RNA was subsequently purified using Qiagen’s RNeasy Lipid Tissue Mini Kit following manufacturers instructions.

### Quantitative PCR

Purified RNA was DNaseI treated with TURBO DNA-*free* Kit (Ambion) and quantified by NanoDrop. 400 ng RNA was used in the first-strand cDNA synthesis reaction primed with random hexamers using Invitrogen’s SuperScript III First-Strand Synthesis SuperMix. cDNA was diluted 1:10 in dH_2_O and 2.5 μl (striatum, hippocampus) or 1.5 μl (cortex) used per 10 μl real-time PCR reaction with 2x LightCycler 480 SYBR Green I Master and 0.5 μM of each primer. Primer sequences used for *Gm12695* were: *Gm12695 F* 5′ cagagcttctgctgatttgtca 3′, *Gm12695 R* 5′ cagggatgtcaaccaagtcc 3′, and for the reference gene *Atp5b*^21^: *Atp5b F* 5′ tgagagaggtcctatcaaaacca 3′, *Atp5b R* 5′ caccagaatctcctgctcaac 3′. Reverse transcriptase negative controls were included for each sample and no-template controls performed for each primer pair. Samples were run in triplicate on a Roche LightCycler 480 2.0 Real-Time PCR System. After an initial denaturation at 95 °C for 5 minutes samples underwent 45 cycles of amplification at 95 °C for 15 seconds and 60 °C for 1 minute, with melting curve analysis performed at the end of each run. Standard curves were run for each primer pair in each tissue group and subsequently used in the relative quantification of *Gm12695*, using the Pfaffl method^8^. *Gm12695* crossing point values for all wild-type samples were at least 5 cycles higher than transgenic samples (with all exceeding 32 cycles), and arbitrary values of 40 were given to those that did not express any *Gm12695.* Therefore, *Gm12695* expression was quantified in transgenic animals by comparison with the combined wild-type mean.

### RNA sequencing

Six RNA-sequencing libraries were prepared from cortical tissue extracted from three R6/2 transgenic female mice and three control mice (age, sex and strain matched from The Jackson laboratory) with a customized version of the strand-specific dUTP method^22^,^23^,^24^, as we have previously described^25^. One microliter of a 1:10 dilution of External RNA Controls Consortium (ERCC) spike-ins (Ambion) containing 92 synthetic RNA standards of known concentrations and sequence was added to each RNA-sequencing library to estimate the expression threshold to detect the expressed genes. Libraries were multiplexed, pooled and sequenced on Illumina HiSeq2000, generating an average of 34.4 million paired-end reads of 51 bp.

### R6/2 Bioinformatic analysis

Quality of sequence reads was assessed by fastQC (v. 0.10.1)^26^. The transgene sequence of 2,463 nt. (GenBank:KF990992.1) was inserted into a mouse reference genome Ensembl GRCm38 (v. 75) by replacing the 5,444 bp-long segment spanning chr4:96,742,795–96,748,238. Sequence reads were then aligned to this modified mouse genome with an inserted transgene sequence using GSNAP (v. 12–19–2014) at its default parameter setting^27^. Quality checking of alignments was assessed by custom scripts utilizing Picard Tools (http://broadinstitute.github.io/picard/), RNASeQC^28^, RSeQC^29^ and SamTools^30^. Genome-guided transcriptome assembly for chromosome 4 was performed using Trinity (v 2.0.6) on combined alignments of all three R6/2 samples with parameters –SS_lib_type RF and –genome_guided_max_intron 10000^31^. Gene level counts were tabulated using BedTools’s multibamcov algorithm (v. 2.17.0)^32^ on unique alignments for each library at all Ensembl genes (GRCm38 v. 75) except *Gm12695*, for which a transcript predicted by Trinity was used. This transcript consisted of a portion of the transgene and exons 8–11 of *Gm12695*. Analysis of ERCC spike-ins as described in Blumenthal *et al*.^33^ estimated the expression threshold for detection to be at least two uniquely mapped reads. 19,514 genes that met this threshold in either all the control samples or all the R6/2 samples were further considered for differential expression analysis, which was carried out using nbinomTest function in an R/Bioconductor package, DESeq (v. 1.18) in R platform (v. 3.1.0). Over-representation analysis of the pathway and gene ontology (GO) terms was performed using DAVID (v. 6.7)^34^, where we used ensemble ids to represent genes and 19,514 expressed genes as a background gene set. Only terms with Benjamini-adjusted p value < 0.05 were reported. Alignments and splice junctions were visualized using the Integrative Genomics Viewer (v. 2.3.72)^35^.

### Htt knock-in mice bioinformatic analysis

RNA sequence datasets for the *Htt* knock-in allelic series are available at NCBI GEO accession numbers: GSE73468, GSE73503, GSE73508, GSE65770, GSE65772, GSE65774, GSE65775, GSE65776. Trimmed fastq files were obtained and aligned to the mouse GRCm38.84 reference genome with STAR^36^ and quantitated at the gene level with HTSeq^37^. These counts were used to calculate FPKM and transcripts per million (TPM) thereby normalizing for both gene size and library size.

## Additional Information

**How to cite this article**: Jacobsen, J. C. *et al*. Potential molecular consequences of transgene integration: The R6/2 mouse example. *Sci. Rep.* **7**, 41120; doi: 10.1038/srep41120 (2017).

**Publisher's note:** Springer Nature remains neutral with regard to jurisdictional claims in published maps and institutional affiliations.

## Supplementary Material

Supplementary Figures

Supplementary Tables

## Figures and Tables

**Figure 1:**
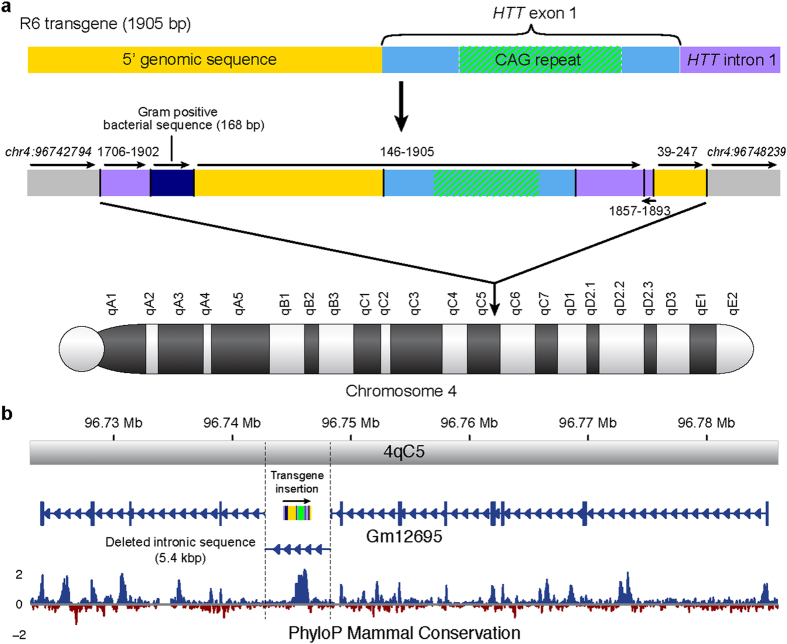
Transgene rearrangement and insertion into the mouse genome. (**a**) The original structure of the R6 transgene (top schematic, drawn to scale) and its extensive resultant rearrangement following integration into chromosome 4 of the R6/2 mouse (bottom schematic) are shown. The R6/2 integrated transgene contained fragments derived from at least three copies of the original R6 transgene, including complex inversions as well as head-to-tail and tail-to-head concatenations. Additionally, a 168 bp fragment of bacterial sequence was integrated upstream of the transgene *HTT* 5′ genomic sequence^2^. (**b**) Integration of a 1.9 kb *HTT* transgene fragment into intron 7 of mouse locus *Gm12695* and concomitant 5.4 kb deletion of intron 7 at the integration site (demarcated by dotted lines). PhyloP^38^ analysis of mammalian conservation spanning the gene is provided, revealing the deleted intronic segment to be among the most conserved regions in the gene, including its coding sequence.
