# Supplementary material for: Potential molecular consequences of transgene integration: The R6/2 mouse example
Source: Sci Rep. 2017 Jan 25;7:41120. doi: 10.1038/srep41120 (PMC5264158; doi:10.1038/srep41120)

Potential molecular consequences of transgene integration: The R6/2 mouse example

Jessie C. Jacobsen, Serkan Erdin, Colby Chiang, Carrie Hanscom, Renee R. Handley, Douglas D. Barker, Alex Stortchevoi, Ian Blumenthal, Suzanne J. Reid, Russell G. Snell, Marcy E. MacDonald, A. Jennifer Morton, Carl Ernst, James F. Gusella, Michael E. Talkowski

Supplementary Figure S1: Dysregulation of *Gm12695* expression in R6/2 heart and liver

Reverse transcription PCR demonstrating *Gm12695* expression in heart and liver from R6/2 mice with 111 and 714 CAG repeats and their littermate controls. R6/2 mice express *Gm12695* in both peripheral tissues, while their littermate controls show negligible expression (a). *Atp5b* was used to validate the quality of the RNA (b). Reverse transcriptase negative controls (RTC) were included for each sample and no-template controls (NTC) performed for each primer pair. RNA extraction, first-strand synthesis and primer sequences were as described in the methods section for quantitative PCR.

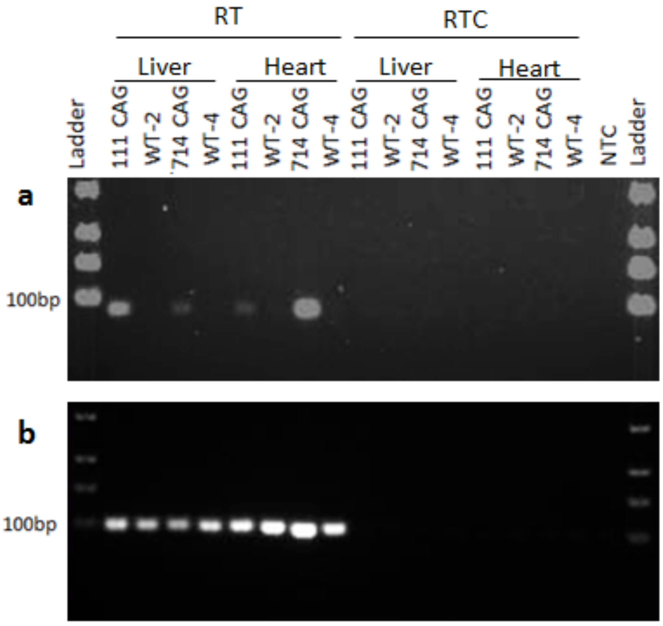

Supplementary Figure S2: Integrated Genome Viewer (IGV) display of RNA sequencing data from R6/2 and control libraries on *Gm12695* sense strand

Top and middle panels illustrate the combined RNA sequencing data as read depth coverage and splice junctions from all three R6/2 libraries and all three control libraries along the sense strand of *Gm12695* respectively. Numbers on the arcs illustrate the reads covering splice junctions. Maximum read depth coverage is 89. The bottom panel illustrates annotated transcripts including a novel transcript predicted by Trinity (predicted transcript) as a result of transgene integration, the known *Gm12695* transcript and the R6/2 transgene at the integration site. The chromosomal coordinates are derived from Supplementary Table S1.

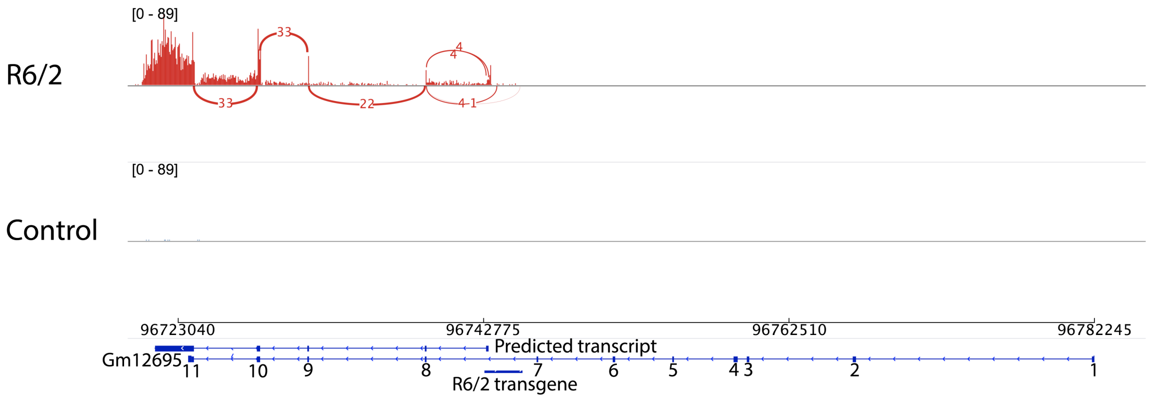

Supplementary Figure S3: IGV display of RNAseq data from R6/2 and control libraries on *Gm12695* antisense strand

Top and middle panels illustrate the combined RNA sequencing data as read depth coverage from all three R6/2 libraries and all three control libraries along the antisense strand of *Gm12695* (transgene sense strand) respectively. Maximum read depth coverage is 504. The bottom panel illustrates annotated transcripts including the known *Gm12695* transcript and the R6/2 transgene at the integration site. The chromosomal coordinates are derived from Supplementary Table S1.

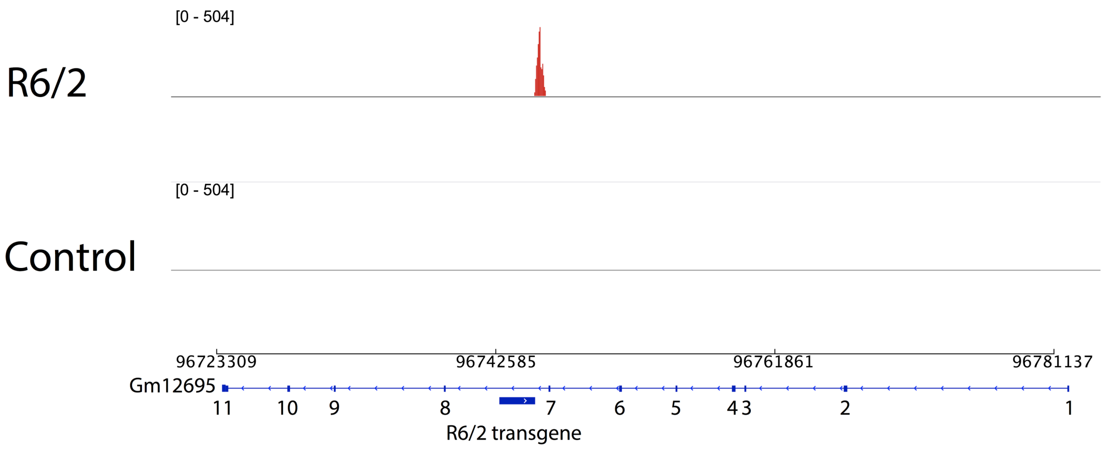

Supplementary Figure S4: IGV display of RNAseq from R6/2 libraries surrounding the transgene on *Gm12695* sense strand

The alignment track illustrates the combined RNA sequencing data as alignments of sequence reads from all three R6/2 libraries along the R6/2 transgene (antisense strand) and flanking regions on the sense strand of *Gm12695* respectively. Light blue lines depict aligned split reads between exon 8 and the R6/2 transgene, whereas grey dots represent the remaining aligned reads on the reverse strand. Reads that are mapped to the forward and reverse strands are depicted in red and blue respectively. The annotation track illustrates annotated transcripts including the known *Gm12695* transcript, a novel transcript predicted by Trinity (predicted transcript) as a result of transgene integration and the R6/2 transgene at the integration site. The chromosomal coordinates are derived from Supplementary Table S1.

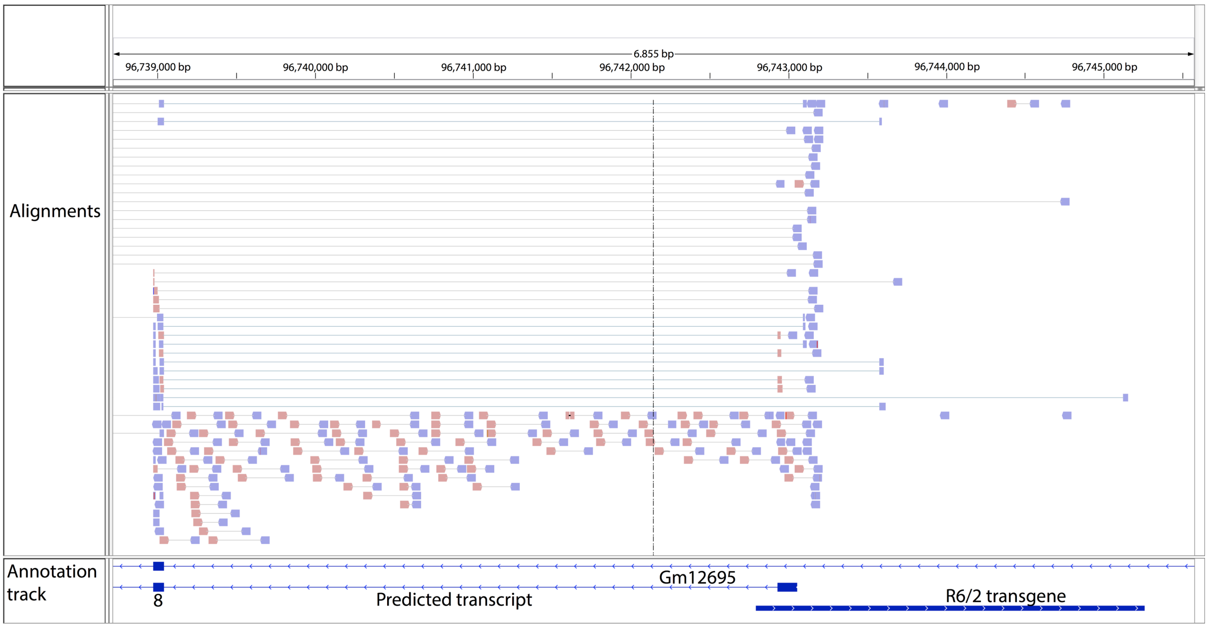

Supplementary Figure S5: IGV display of RNAseq from R6/2 libraries surrounding the transgene on *Gm12695* antisense strand

Top and middle panels illustrate the combined RNA sequencing data as read depth coverage and alignments of sequence reads from all three R6/2 libraries along the R6/2 transgene and downstream region on the sense strand of R6/2 transgene respectively (*Gm12695* antisense strand). Grey lines connect the aligned read pairs, where reads that are mapped to the forward and reverse strands are depicted in red and blue respectively. A peak in the coverage track illustrates significant accumulation of mapped reads on repeat elements. Maximum read depth coverage is 504. The bottom panel illustrates annotated transcripts including the known *Gm12695* transcript, the R6/2 transgene at the integration site and locations of a long terminal repeat, MLT1A on the reverse strand and a long interspersed nuclear element, L1Md_T on the forward strand. The chromosomal coordinates are derived from Supplementary Table S1.

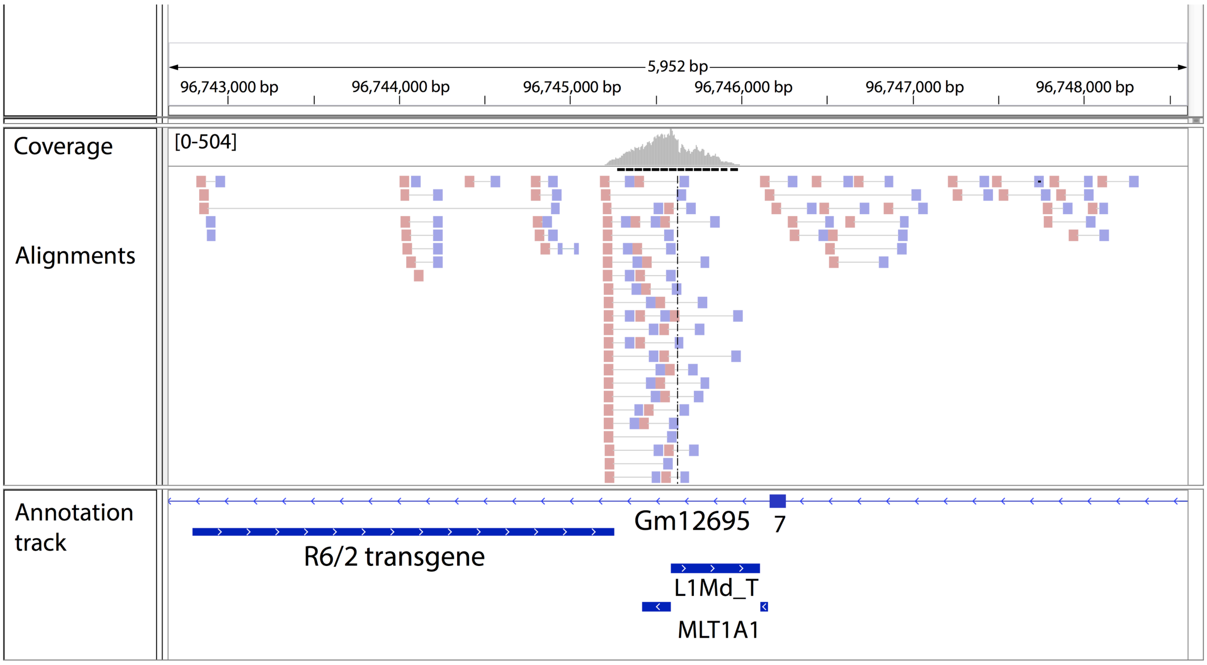

Supplementary Figure S6: ***Gm12695*** **mRNA expression in five HD mouse models across five tissues and three ages**

Box plots showing *Gm12695* (blue) and *Atp5b* mRNA (red) levels (RNAseq transcripts per million) in cerebellum, hippocampus, striatum and liver from heterozygous *Htt* CAG repeat knock-in mice with different sized CAG repeat tracts (Q20, Q80, Q92, Q111, Q140, Q175) at 2 months (top panel), 6 months (middle panel) and 10 months (bottom panel). Each box plot is derived from 7-9 samples.

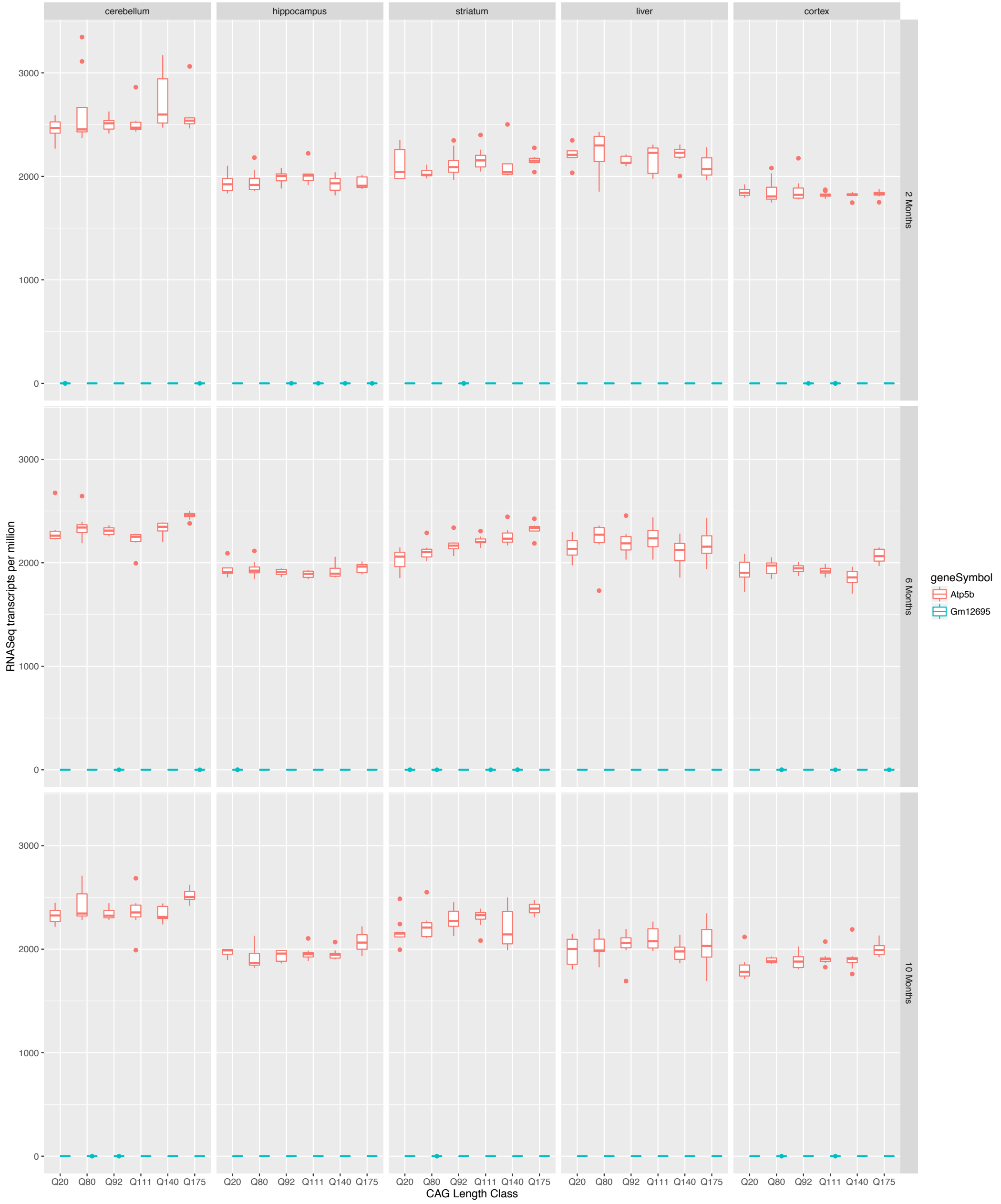

Supplement: Supplementary Figures [file srep41120-s1.zip › Jacobsen Supp Figures SREP-16-38239.pdf]
